# The earliest known crown-*Testudo* tortoise from the late Miocene (Vallesian, 9 Ma) of Greece

**DOI:** 10.1371/journal.pone.0224783

**Published:** 2020-04-08

**Authors:** Géraldine Garcia, Aurélie Pinton, Xavier Valentin, Dimitris S. Kostopoulos, Gildas Merceron, Louis de Bonis, George D. Koufos

**Affiliations:** 1 PALEVOPRIM, UMR CNRS 7262, Université de Poitiers, Poitiers, France; 2 Department of Geology, Aristotle University of Thessaloniki, Thessaloniki, Greece; Eberhard Karls Universitat Tubingen, GERMANY

## Abstract

We here report on fossil remains of the earliest known crown-*Testudo*, an extant clade of Mediterranean testudinid tortoises from the late Miocene (Vallesian, MN 10) from the hominoid locality Ravin de la Pluie (RPl) in Greece. The material studied is a small, nearly complete carapace with a clearly distinct hypo-xiphiplastral hinge. This supports the *sensu stricto* generic assignment. This new terrestrial testudinid specimen is characterized by a possible tectiform, narrow, elongated shell with a pentagonal pygal and a long, posteriorly elevated, lenticular and rounded dorsal epiplastral lip. These unique features differ from those of other known Mediterranean hinged forms and allow the erection of the new species *Testudo hellenica* sp. nov. This taxon is phylogenetically close to two Greek species, the extant *T*. *marginata* and the fossil *T*. *marmorum* (Turolian, around 7.3 Ma). This record provides evidence for the first appearance of the genus *Testudo sensu stricto* at a minimum age of 9 Ma.

## Introduction

**Fig 1:**
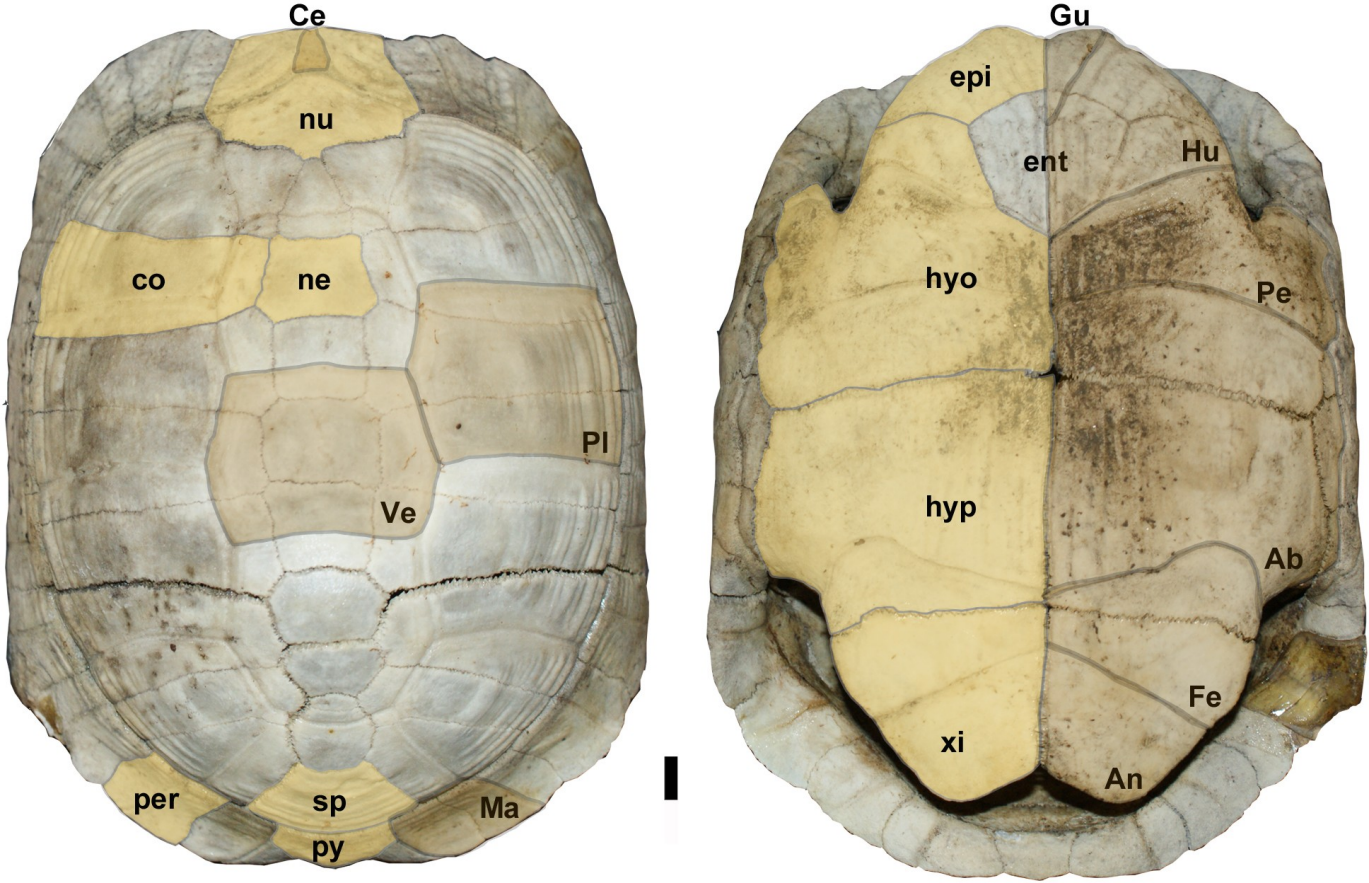
Extant *Testudo graeca* (UP-2015-05) from Greece. nu: nuchal; ne: neural; co: costal; sp: suprapygal; py: pygal; per: peripherals; epi: epiplastron; ent: entoplastron; hyo: hyoplastron; hyp: hypoplastron; xi: xiphiplastron. Scutes: Ce: cervical; Pl: pleural; Ma: marginal, Ve: vertebral; Gu: gular; Hu: humeral; Pe: pectoral; Ab: abdominal; Fe: femoral; An: anal.

*Testudo sensu stricto* (s. s. i.e., *Testudo* with a hinge) is defined and restricted to the small tortoise species having a kinetic hinge in its plastron between the hypo- and xiphiplastra (Fig 1) [1, 2]. Based on molecular data [3–7], the *Testudo s*. *s*. group is monophyletic and restricted to the three extant tortoise species distributed geographically between the Mediterranean region, Caucasus and Iran: *Testudo graeca* [8] (Fig 1), *Testudo marginata* [9] and *Testudo kleinmanni* [10]. This Mediterranean group is supported by a total evidence analysis (morphological and molecular data) as the crown clade arising from the last common ancestor of *T*. *graeca*, *T*. *marginata* and *T*. *kleinmanni*, estimated between 8 to 7 Ma following Vlachos & Rabi [11]. The oldest attested fossil with a hypo-xiphiplastral hinge is *Testudo marmorum* [12, 13] from the classic Pikermi beds, a Turolian locality situated in the North Aegean region near Athens in southern Greece (MN 12) dated from 7.35 to 7.28 Ma by Böhme et al. [14].

**Fig 2:**
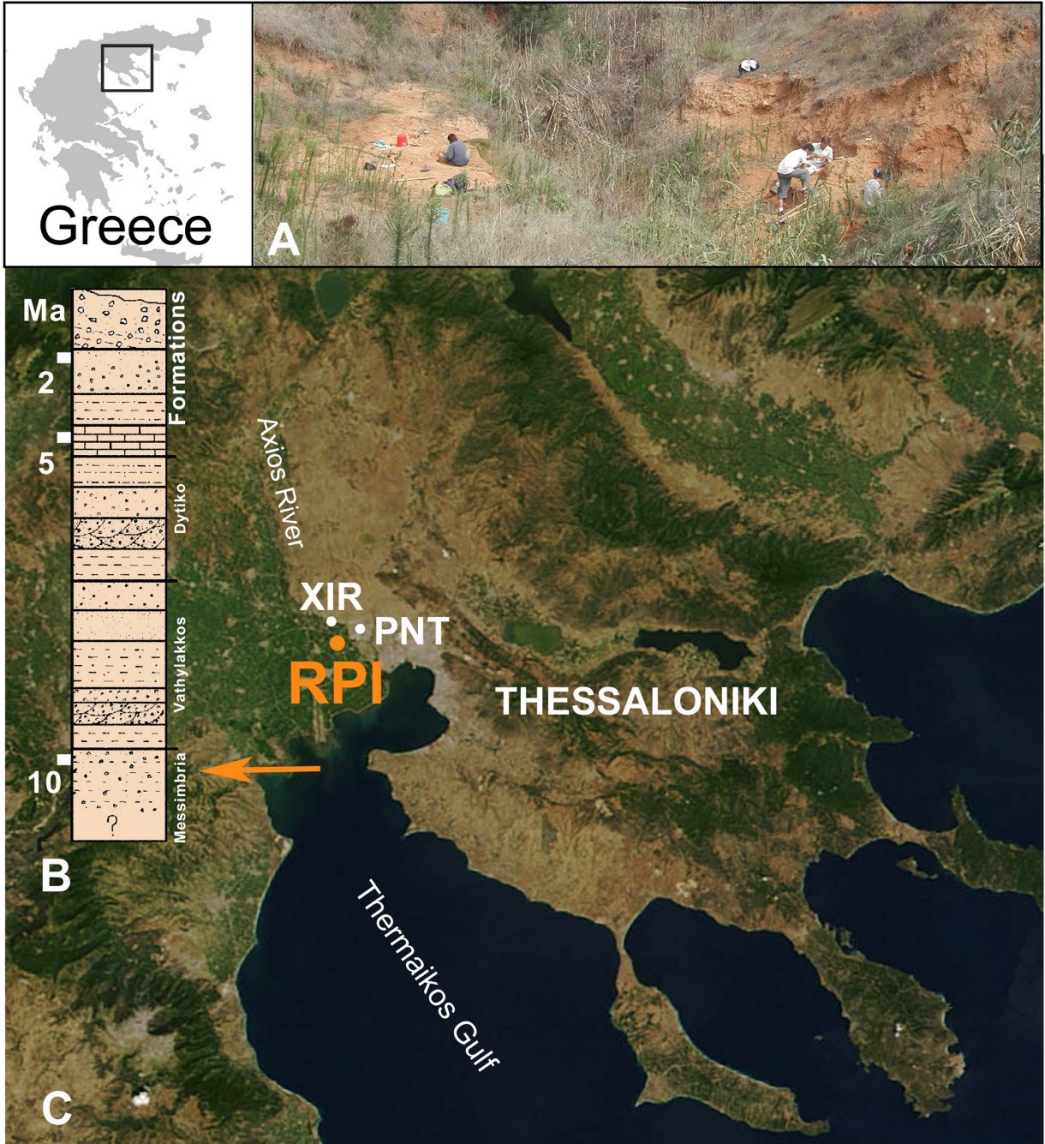
The late Miocene site of Ravin de la Pluie, RPI (Thessaloniki Macedonia, northern Greece). View of the dig (A) and its location of the stratigraphy modified after [19] (B) and in the Axios Valley (C), near the two other richest Vallesian vertebrate localities. XIR: Xirochori and PNT: Pentalophos. Source of the satellite image: NASA Earth Observatory.

A new species of tortoise is described here, that corresponds the earliest *Testudo* s. s. known in the fossil record. It is composed of an almost complete carapace and represents the only small tortoise coming from the deposits of the Axios Valley in northern Greece. Historically, the mammal fossiliferous sites of the Axios Valley (Macedonia, Greece) have been known since the beginning of the 20^th^ Century when the paleontologist Camille Arambourg, an officer in the French Army during the First World War at that time, discovered several localities with his soldiers “the Zouaves” and collected some vertebrates still housed today in the Muséum National d’Histoire Naturelle de Paris, France [15]. Decades later, new fieldwork campaigns were reinitiated and have been conducted since the seventies [16]. Numerous outcrops have been revealed in a small perimeter along the Axios Valley from the Vallesian Nea Messimbria Formation to the Turolian Vathylakkos and Dytiko Formations [17]. Among them, Pentalophos (PNT), Xirochori (XIR), and Ravin de la Pluie (RPI) are the three main localities of Vallesian age with the richest faunal assemblages of large mammals (MN 10 9.7 to 8.7 Ma, [18]) (Fig 2). The latter site corresponds to the type locality of the hominid *Ouranopithecus macedoniensis* (see example [19–23]).

While both mammalian diversity and abundance are exceptional in the Vallesian sites, with at least 24 mammal species in RPI [24], reptile remains (lizards and snakes) are scarce. Few terrestrial testudinid specimens were discovered during the numerous field missions of these last decades. Although they are known since the nineteenth century and abundant in Miocene Greek sediments, data on the Testudinidae were limited in the literature, often based on poorly preserved or not formally identified specimens [25] until recently with new published data [26, 27]. There are a few known examples of tortoise remains from the middle Miocene of Chios Island (Keramaria Formation, [28]) but they have not been illustrated or affiliated to the modern *Testudo* [29]. All other Neogene testudinid fossils from Greece are more recent, from the Turolian (MN 11, around 8 Ma) to the Villafranchian (MN 16, 3 Ma) [30, 31].

## Material and methods

**Fig 3:**
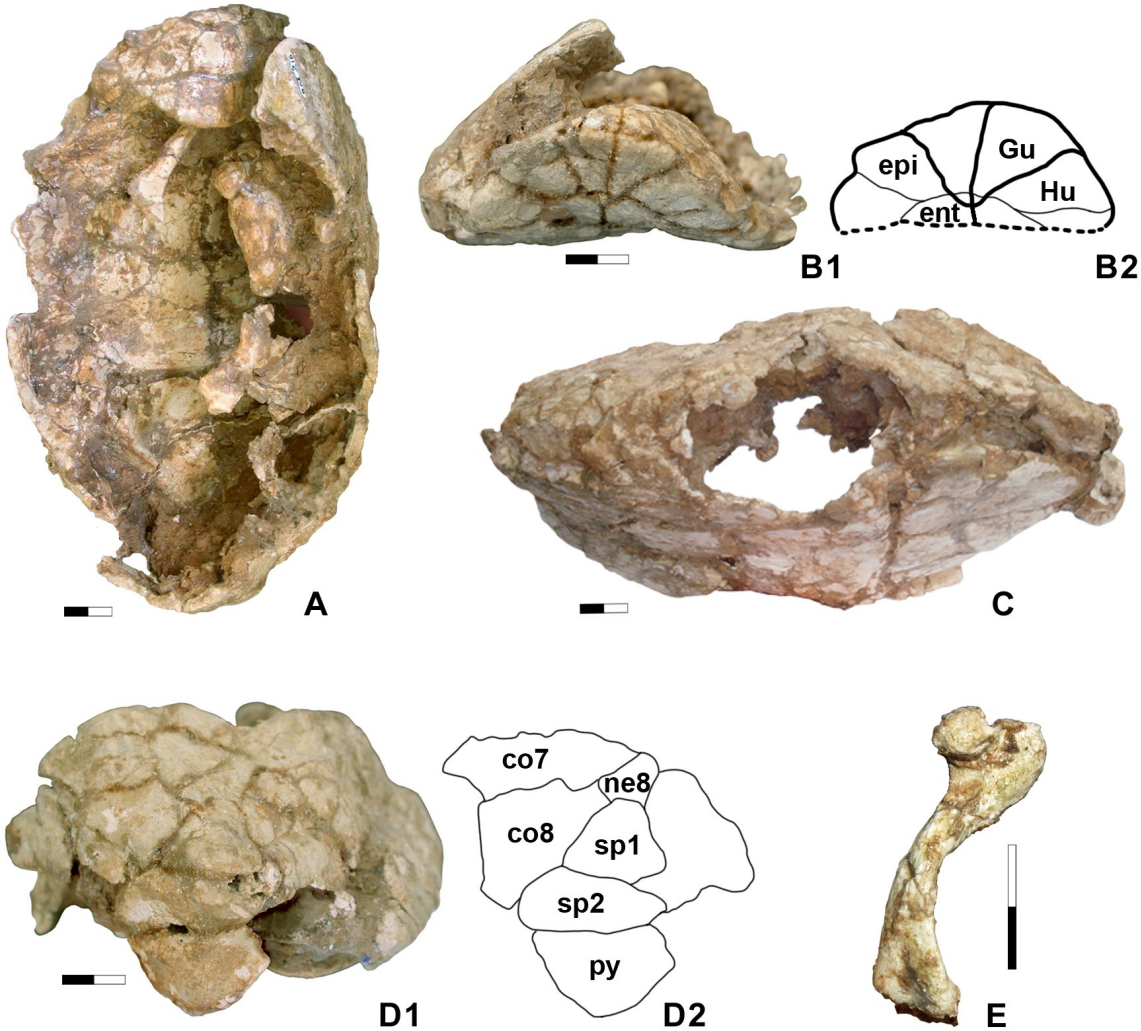
*Testudo hellenica* sp. nov. from Ravin de la Pluie (Greece, late Miocene). Holotype, LGPUT RPl 16. A. Carapace in dorso-lateral view. Note the very obtuse scapula and the deep nuchal notch. B1. Anterior plastral lobe. B2. Interpretative drawing. C. Right lateral view of the carapace. D1. Posterior shell border with suprapygal-pygal configuration. D2. Interpretative drawing. E. Left humerus in lateral view. Scale: 2 cm.

**Fig 4:**
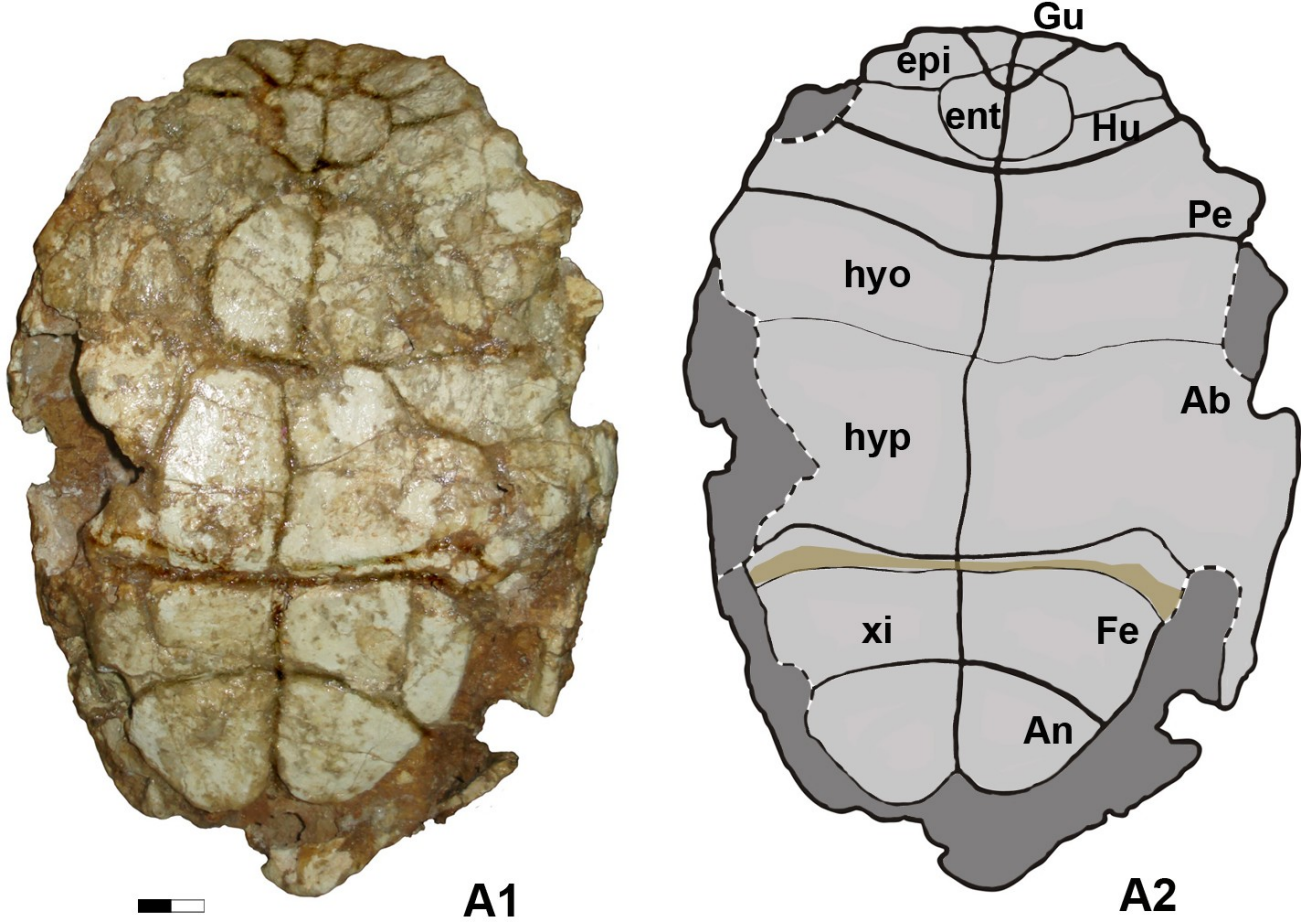
A1. Plastron in ventral view. A2. Interpretative drawing.

The carapace (RPI-216) was collected in the 1980s in the Ravin de la Pluie (RPI), located in the Nea Messimvria Formation (Fig 2). This corresponds to a succession of sands, gravels, conglomerates and reddish clays, reflecting fluviatile paleoenvironments [17]. The carapace with plastron is well preserved with relatively low deformations (Figs 3 and 4). In order to observe the detail of the anatomical elements, the specimen was completely prepared. It was found in association with numerous vertebrate fossil remains, including diversified large mammal taxa dominated by herbivorous taxa such as bovids (*Samotragus praecursor*, *Prostrepsiceros vallesiensis*, *Mesembriacerus melentisi* and *Palaeoryx* sp.), giraffids (*Palaeogiraffa macedoniae*, *Palaeotragus* cf. *coelophrys* and *P*. cf. *rouenii and Bohlinia* cf. *attica*), equids (*Hipparion primigenium*, *H*. *macedonicum* and *H*. aff. *depereti*) and rhinocerotids (Rhinocerotidae indet.).

In order to strengthen the systematic attribution of the fossil and discuss its affinities, a phylogenetic analysis was performed based on a published morphological matrix (Table 1), which includes 20 taxa and 37 characters [32] that helped to resolve the relationships between the various *Testudo* s. l. based on morphological characters. The definition and the coding of some characters were modified (S1 Table). The phylogenetic analyses were performed with PAUP (Phylogenetic Analysis Using Parsimony) version 4.0b10 [33] using the branch and bound algorithm. Following Lujan et al. [34] all characters are treated as unordered and unweighted, branches were set to collapse if minimum length equals zero. However, to ensure the robustness of our topology we performed a run in which multistate characters were ordered. Tree topologies resulting from ordered *versus* unordered analysis (data not shown) are identical. Clade robusticity was assessed by means of bootstrap analysis (10000 replicates) and Bremer support indices, which have been calculated by hand running consecutive analysis and holding trees with one step more each time.

**Table 1:** Data matrix of the cladistic analysis, modified after Lujan et al [32]. Missing data are represented by a question mark. “A” equals (0,1).

**Taxon character**	1	2	3	4	5	6	7	8	9	10	11	12	13	14	15	16	17	18	19	20	21	22	23	24	25	26	27	28	29	30	31	32	33	34	35	36	37
*Malacochersus tornieri*	0	0	0	0	0	0	0	0	0	0	0	0	0	0	0	0	0	0	0	0	0	0	0	0	0	0	0	0	0	0	0	1	1	1	1	0	0
*Indotestudo elongata*	0	0	0	0	0	0	0	0	0	0	0	0	0	0	2	0	0	0	0	0	0	0	0	0	0	0	0	0	0	1	0	0	2	1	1	0	0
*Testudo Testudo hellenica*	1	1	?	1	?	0	?	0	?	?	1	0	0	1	2	0	0	?	0	?	0	0	?	2	3	0	?	0	?	0	1	1	0	0	0	1	?
*T*. *(Testudo) graeca*	1	1	0	1	A	0	2	0	0	0	0	0	0	1	1	0	0	0	0	0	0	0	1	0	3	0	1	0	0	1	1	1	1	0	2	0	0
*T*. *(Testudo) marginata*	1	1	0	1	0	0	2	1	0	0	1	0	0	1	2	0	0	1	0	0	0	1	1	0	3	0	1	1	0	1	1	1	1	0	2	A	1
*T*. *(Testudo) marmorum*	1	1	?	1	0	0	?	1	0	0	1	0	0	1	2	0	0	0	0	0	0	1	0	0	3	0	1	0	0	0	1	1	0	0	2	1	1
*T*. *(Testudo) kleinmanni*	1	1	0	1	0	0	2	0	0	0	1	1	0	1	1	0	0	0	1	0	0	0	A	0	3	0	1	0	0	0	1	1	0	0	2	1	0
*T*. *(Testudo) kenitrensis*	1	1	?	1	0	0	2	0	0	0	1	1	1	1	1	0	0	1	0	0	0	0	0	0	3	0	1	0	1	?	1	1	0	0	2	1	0
*T*. *(Testudo) oughlamensis*	1	1	?	1	0	0	?	0	0	0	1	1	1	1	1	0	0	0	0	0	0	0	0	0	3	0	1	0	0	0	1	A	0	0	2	1	0
*T*. *(Testudo) brevitesta*	1	?	?	?	?	0	?	1	?	0	?	?	?	1	3	0	?	?	1	?	0	?	0	0	3	0	1	1	?	?	1	1	0	0	?	?	?
*T*. *(Agrionemys) horsfieldii*	1	1	0	0	0	1	1	0	0	1	0	0	0	1	3	0	0	1	0	0	0	0	1	0	2	1	1	0	1	0	1	0	2	0	0	1	0
*T*. *(Agrionemys) bessarabica*	1	?	?	0	0	?	?	0	0	?	0	0	0	1	3	0	0	1	0	0	0	0	0	1	2	0	?	0	?	0	1	?	1	0	0	0	0
*T*. *(Chersine) canetoniana*	1	1	1	0	0	0	?	0	1	1	0	0	0	1	1	0	0	0	0	1	0	0	0	0	2	0	1	0	1	1	1	0	0	0	0	0	0
*T*. *(Chersine) hermanni*	1	1	1	0	1	0	1	0	1	1	0	0	0	1	1	0	1	0	1	1	1	0	A	1	2	1	1	0	1	1	1	0	1	0	0	1	0
*T*. *(Chersine) steinheimensis*	1	1	?	0	1	0	?	0	1	1	0	0	0	1	1	1	0	0	0	1	0	0	A	0	1	0	1	0	1	0	1	A	A	0	0	0	0
*T*. *(Chersine) catalaunica*	1	1	1	0	1	0	?	0	1	1	0	0	0	1	1	1	0	0	0	1	0	0	0	0	2	0	1	0	1	A	1	A	A	0	0	0	0
*T*. *(Chersine) antiqua*	1	1	?	0	1	0	?	0	1	1	0	0	0	1	1	0	1	0	0	1	0	0	A	0	2	0	1	0	1	A	1	A	0	0	0	0	0
*T*. *(Chersine) burgenlandica*	1	1	1	0	1	0	?	0	1	1	0	0	0	1	1	0	1	2	0	1	0	0	0	0	2	0	1	0	1	0	1	0	0	0	0	0	0
*T*. *(Chersine) pyrenaica*	1	1	1	0	1	0	?	0	1	1	0	0	0	1	1	0	?	2	0	1	A	0	0	0	2	1	1	0	1	1	1	0	0	0	0	0	0
*T*. *(Chersine) lunellensis*	1	1	1	0	1	0	?	0	1	1	0	0	0	1	1	0	1	0	A	1	1	0	0	1	3	A	1	0	1	0	1	0	0	0	0	0	0

The specimen is stored in the collections of the Geology and Palaeontology laboratory of the Aristotle University of Thessaloniki (LGPUT), accession number RPI-216.

### Nomenclatural acts

The electronic edition of this article conforms to the requirements of the amended International Code of Zoological Nomenclature, and hence the new names contained herein are available under that Code from the electronic edition of this article. This published work and the nomenclatural acts it contains have been registered in ZooBank, the online registration system for the ICZN. The ZooBankLSIDs (Life Science Identifiers) can be resolved and the associated information viewed through any standard web browser by appending the LSID to the prefix "http://zoobank.org/". The LSID for this publication is: urn:lsid:zoobank.org:pub:B6A6B0A2-57F9-4EB6-9C09-CB50DAB1BDB3. The electronic edition of this work was published in a journal with an ISSN, and has been archived and is available from the following digital repositories: PubMed Central, LOCKSS.

### Systematic paleontology

Cryptodira Cope, 1868

Testudinidae Batsch, 1788

*Testudo* Linnaeus, 1758

*Testudo hellenica* sp. nov. (Figs 3 and 4)

*Testudo hellenica* Garcia et al. sp. nov. urn:lsid:zoobank.org:act:3036B41A-88AE-4BE5-ADEE-C7F4C11AC0A1

The name of this new species was already mentioned at the congress communication [34], but it must be considered as a *nomen nudum* because the species was not described according to the rules of the ICZN code.

**Holotype.** LGPUT RPI-216, a nearly complete carapace with plastron.

**Etymology.** Named after the Greek word “ελληνικη” (hellenic, adj. of Hellas = Greece) meaning “from Greece”

**Type locality.** Ravin de la Pluie, lower Axios Valley, Macedonia, Greece; late Miocene (Vallesian Mammal Age, Zone MN 10, correlated with chron C4AR.1N, 9.069–9.149 Ma based on the combination of the evolutionary grade of the mammalian fauna and paleomagnetostratigraphy [35].

**Diagnosis.** Species of *Testudo* s. s. by the hypo-xiphiplastral hinge, characterized by the autapomorphic features of an elongated and quite posteriorly pointed shell shape, with a pentagonal pygal protruding downwards relative to the posterior peripheral border and having a short suture with a peripheral 11, and a lenticular, posteriorly long dorsal epiplastral lip, rounded from side to side and anteriorly to posteriorly convex. Autapomorphies: posterior lobe comprised only by the xiphiplastra with very rounded lateral borders lacking femoro-anal inflexion and with anals slightly medially longer than femorals but without an angular junction of the right and left femoro-anal sulci.

**Description.** The specimen corresponds to a small, nearly complete carapace, (dimensions: 133 x 228 mm: width/length = 58.33%) with an elongated shape, though it narrows, becoming at least anteriorly angular, nearly tectiform (Fig 3A and 3B1). It lacks the left and dorsal part of the shell. The right peripherals and marginals are hard to distinguish, due to the state of preservation of the dorsal shell. The anterior peripheral shell border is elongated on each side of the middle, particularly protruding at the junction of peripherals 1 and 2, which are visible on the right side. This suggests a somewhat pronounced nuchal notch, with its right part preserved. At the posterior of the shell (Fig 3D1), which is elongated and posteriorly pointed, the suprapygals (sp) compose two transversally separated trapezoids in front of the pygal. They are distinct in their proportions with sp2 more elongated transversely than sp1. The pygal is pentagonal, strongly protruding downwards in relation to the adjacent peripheral border: peripherals 11 are not preserved between the pygal and peripherals 10. However, we can observe in posterior view that the lateral suture between the pygal and the peripheral 11 is short (Fig 3C and 3D1), thus suggesting that the peripheral 11 was reduced. The pygal has no medial sulcus, indicating the presence of a supracaudal formed by the fusion of both marginals 12. The domed pygal plate, curving inwards, indicates a male individual [36]. The posterior peripherals, shifted slightly externally in relation to the pleurals, are barely visible from a dorsal view, dorsally convex, and are neither elevated nor elongated.

Some bones are preserved inside the carapace, such as the left humerus (Fig 3E) and a scapula. The scapula is wide, presenting an obtuse angle as in all the terrestrial testudinids (Fig 3A). The humerus (Fig 3E) is robust and shows the great trochanter moderately close to the minor one, as in *Testudo* species, delimiting a triangular intertrochanteric fossa. The plastral buttresses, erected in columns inside the shell, are somewhat distinguishable and conform to terrestrial testudinids (Fig 3A).

Ventrally, the plastron (Fig 4A1) shows a longitudinal medial concavity, indicating a male specimen. The rounded anterior lobe (58.8 mm long) is semicircular with a slightly straight anterior gular border that is upwardly tilted, and the posterior lobe is slightly longer (61.6 mm) with well-rounded lateral borders and a short anal posterior notch with straight borders. The bridge between these two lobes is relatively long (102.7 mm). The dorsal epiplastral lip is very pronounced, transversally lenticular and medially long, anteroposteriorly convex, rounded from side to side with a flat intermediate part (Fig 3A). It projects dorsally beyond the anterior part of a small entoplastron marked by a slight and small depression: the gular pocket. Due to the strong epiplastral lip, the epiplastra are anteriorly raised in a ledge above the dorsal surface of the anterior lobe (Fig 3B1). Ventrally, the gulars are triangular and their ventral surfaces appear as flat as that of the humerals. The humero-pectoral sulcus (HP) runs lateroposteriorly behind the entoplastron (Fig 4A1), with no contact, in a wide curve. The axillary and inguinal scutes are both large, possibly trapezoidal in shape and the posterior lobe does not completely fill the caudal shell opening. While anterior to the hypo-xiphiplastral suture, the abdomino-femoral sulcus is very close to it, widely and particularly medially, the suture being transformed into a hinge (Fig 4A1) and the posterior lobe is formed exclusively by the xiphiplastra.

## Discussion

*Testudo hellenica* sp. nov. is a new small tortoise from the Vallesian (late Miocene) of Greece. It represents the earliest well-dated record of the genus *Testudo sensu lato* (s. l.) with robust phylogenetic relationships. The shell of this species has the typical features of the genus, such as the plate and scute proportions and configurations (costal/peripheral sutures coinciding with pleural/marginal sulci, contact between marginal/pleural sulci, suprapygal/pygal configuration with trapezoid sp1 and sp2), the trigonous gulars and the dorsal epiplastral lip which is curved onto the entoplastron. The phylogenetic relationships within this genus have been subject of continuous discussions [2, 4, 30, 37, 38, 39 and 40]. According to the latest multi-locus molecular phylogeny [7], this clade includes the extant *T*. (*Agrionemys*) *horsfieldi*, *T*. (*Chersine*) *hermanni*, the extant hinged species including *T*. *graeca* and *T*. *marginata* and *T*. *kleinmanni* distributed in the Mediterranean basin and Central Asia.

**Fig 5:**
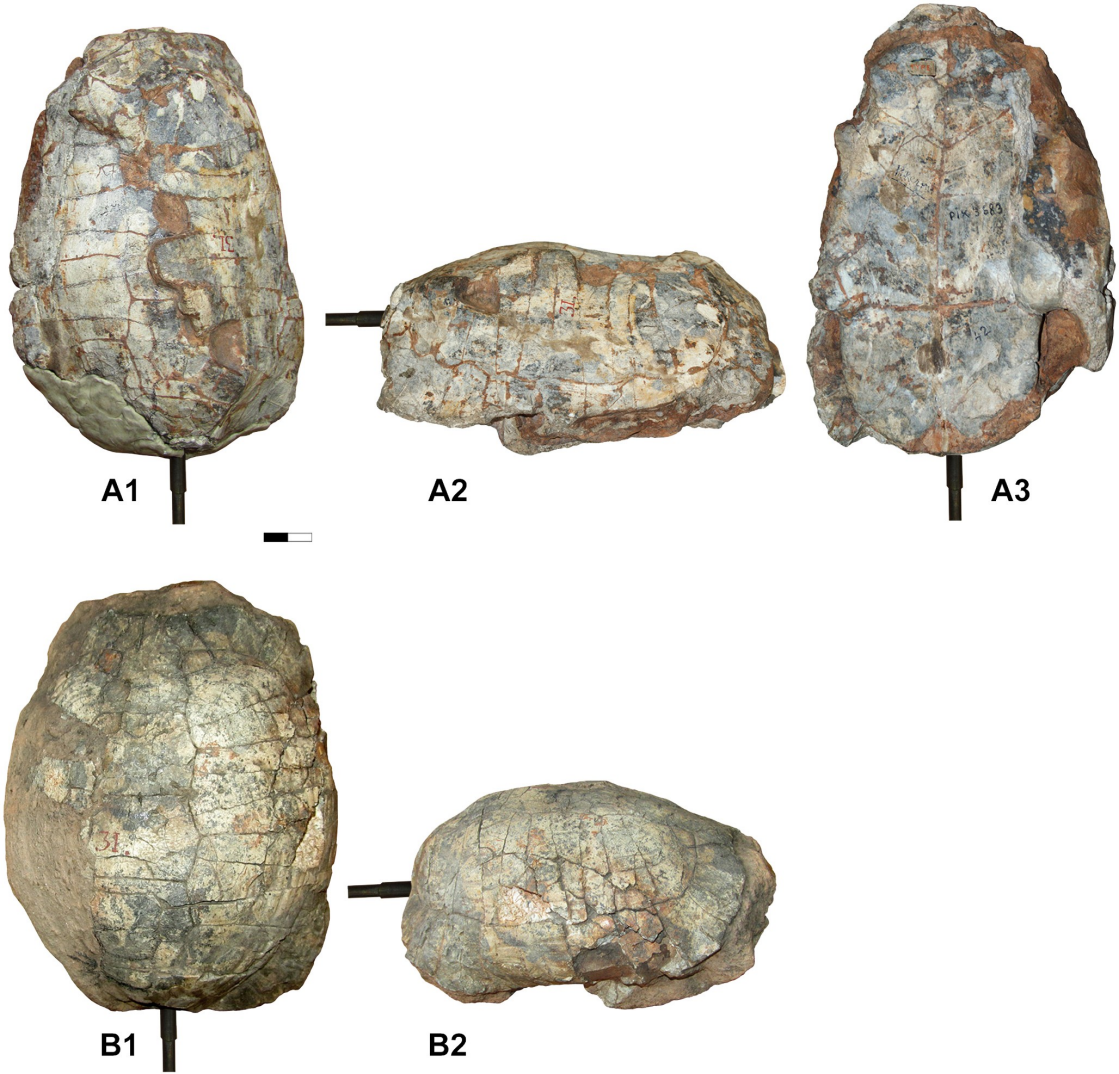
*Testudo marmorum* from Pikermi (Greece, late Miocene) described by Gaudry in 1862, 1862–1867. Holotype MNHN F 1862-67(42) PIK 3683, Carapace in dorsal (A1), lateral (A2) and ventral views. Specimen MNHN F 1862-67(43), Carapace in dorsal (B1) and lateral (B2) views. No plastron is preserved in this specimen.

**Fig 6:**
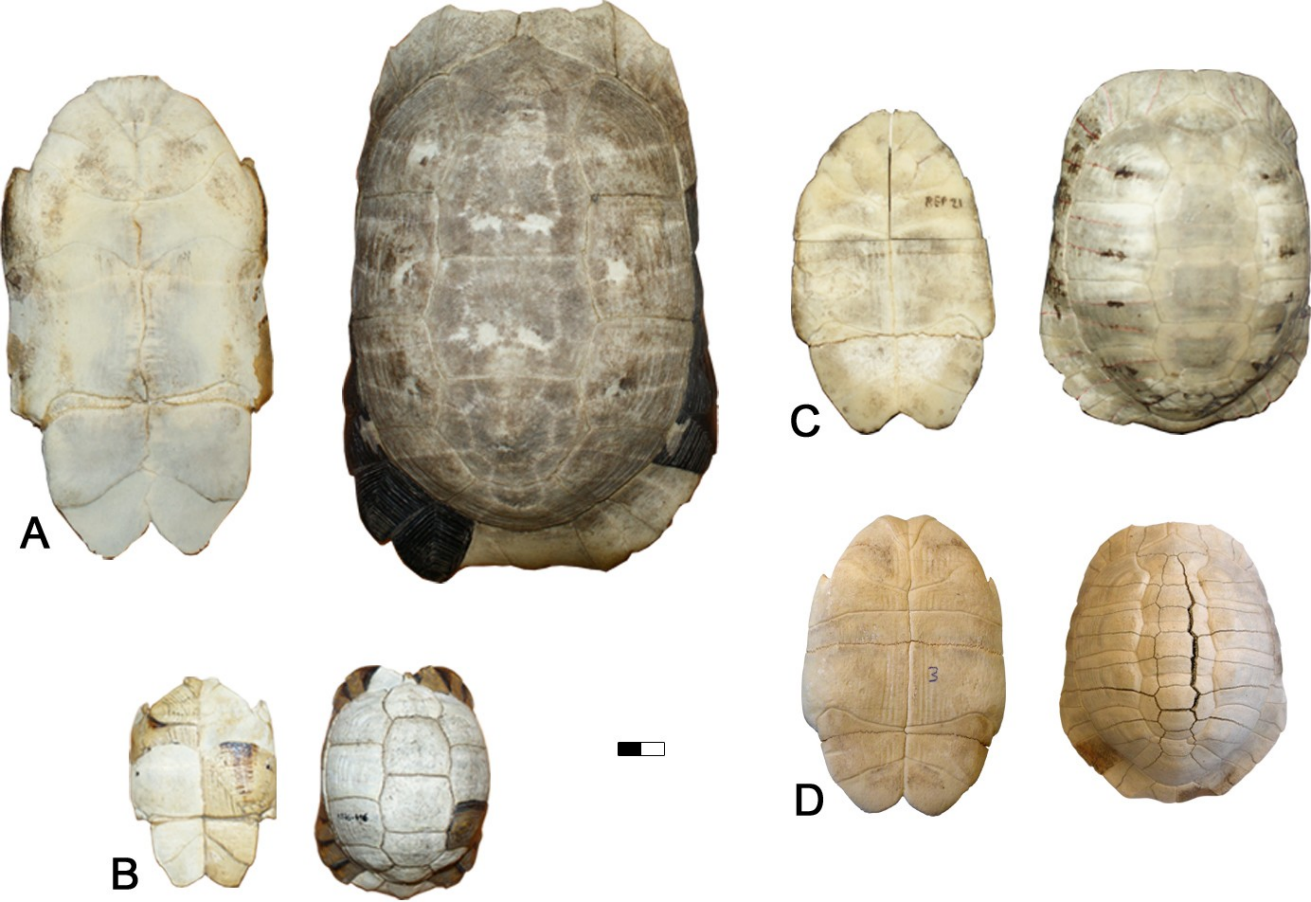
Modern *Testudo*. Carapace in ventral and dorsal views. A. *T*. *marginata*, 1877–675 MNHN. B. *T*. *kleinmanni*, 1876–416 MNHN. C. *T*. *graeca*, REP 21. D. *T*. *hermanni*, REP 7.

By its autapomorphic features, *T*. *hellenica* differs from other species of the *Testudo* s. s. group such as *T*. *graeca*, *T*. *marginata*, *T*. *brevitesta* (Villafranchian, Greece; [30]), *T*. *kleinmanni*, *T*. *kenitrensis* (Pliocene, Morocco; [41]) and *T*. *oughlamensis* (upper Pliocene, Morocco; [42]) and in particular from *T*. *marmorum* (Turolian, Greece) (Figs 5 and 6, [15]). These characters include an angular dorsal shell, not as wide and rounded as that of other species of *Testudo* s. s. and shorter than that of *T*. *marginata* or *T*. *marmorum*; the anteroposteriorly elongated and tilted peripherals on the anterior shell border with a possibly pronounced notch at the nuchal border; a long, lenticular, posteriorly elevated and rounded dorsal epiplastral lip that overhangs the dorsal anterior lobe surface; and a protruding pentagonal pygal. *Testudo hellenica* also differs by the configuration of its posterior plastral lobe with curved rather than rectilinear xiphiplastral borders, lacking parallel borders along the femoral part, as is the case in *T*. *marmorum*, and with curved borders at the femoro-anal sulcus (usually straight and oblique in *Testudo* spp.). The rounded shape of the xiphiplastra is the same as in the short and small Moroccan *T*. *kenitrensis* and *T*. *oughlamensis* but *T*. *hellenica* does not have their angular, less obtuse femoro-anal sulcus. Some characters, such as the shape and the size of the nuchal, the number of neurals and the width of the vertebrals cannot be assessed, due to the state of preservation of the shell. The separation of the xiphiplastra from the hypoplastra indicates the presence of the hinge, allowing movements of the posterior lobe of the plastron. This character, present in both males and females in contrast to *Testudo (Chersine) hermanni*, is a synapomorphy of the *Testudo* s. s group ([2], this study). *Testudo hellenica* shares with *T*. *marginata* and *T*. *marmorum* the elongated shell (ratio of median height and full length in Fig 3A and Fig 3C). The shell dimensions measured for these species are similar to those of all other analysed specimens (Table 2), except for the holotype *T*. *marmorum* (MNHN F 1862-67(42) PIK 3683, Fig 5A1–5A3). This latter is narrower and has a concave plastron, and is different to from the other specimen of *T*. *marmorum* (MNHN F 1862-67(43), Fig 5B1 and 5B2). They correspond respectively to a male and a female, thus conforming with the sexual dimorphism of *T*. *marginata*.

**Table 2:** Measurements of specimens analyzed and included in the comparative study. Fossil taxa are displayed on a grey background.

		Dorsal shell				Plastron				
Specimen and catalog number	Localization	length	width	n3/n4-sp1/sp2 distance	V3 size l/L	Total length	anterior lobe	bridge	posterior lobe	anal notch
*Testudo marmorum* F 1862-67(42) (Type) MNHN	Greece (Upper Miocene)	18.4	11.5	8.2	4/2.9	17.3	4	7.7	5.8	1
*Testudo marmorum* F 1862-67(43) MNHN	Greece (Upper Miocene)	15.8	13	8.5	4/3.0	—	—	—	—	—
*Testudo hellenica* RPI-216 Univ. Thessaloniki	Greece (Upper Miocene)	22.8	13.3	—	—	21.4	5.7	9.8	5.9	1.2
*Testudo graeca antakyensis* REP 50 MNHN	Syria	14.9	11.6	7.5	4.5/3	14.1	3.7	7.4	3	less 1
*Testudo graeca ibera* REP 66 MNHN	Syria	18.6	15	8.6	6/3.8	incomplete	4.3	7.5	—	—
*Testudo graeca ibera* REP 73 MNHN	indeterminate	21	18.8	8.8	6.5/4	17.5	4	8.5	5	0.8
*Testudo graeca graeca* REP 21 MNHN	north of Africa	15.2	11.7	6.7	4.5/2.9	12.9	3.2	6.4	3.3	0.9
*Testudo hermanni* REP 7 MNHN	France	13.4	10.9	6.5	3.3/2.5	12.7	2.8	6.5	3.4	—
*Testudo kleinmanni* 1876–416 MNHN	Egypte	11.1	7.4	4.8	2.9/2.2	9	1.7	4.6	2.5	0.5
*Testudo marginata* 1877–675 MNHN	indeterminate	21.8	12.5	10.2	5.7/3.8	17.5	3.7	9.8	4.7	1.4
*Testudo marginata* 1887–831 MNHN	indeterminate	22.6	13.4	9.4	5.3/3.7	18	3.9	9.6	4.8	1.2
*Testudo marginata* 1878–199 MNHN	indeterminate	26.9	15.5	12.2	6.8/4.5	22.3	5.1	10.7	5.9	2.2

**Fig 7:**
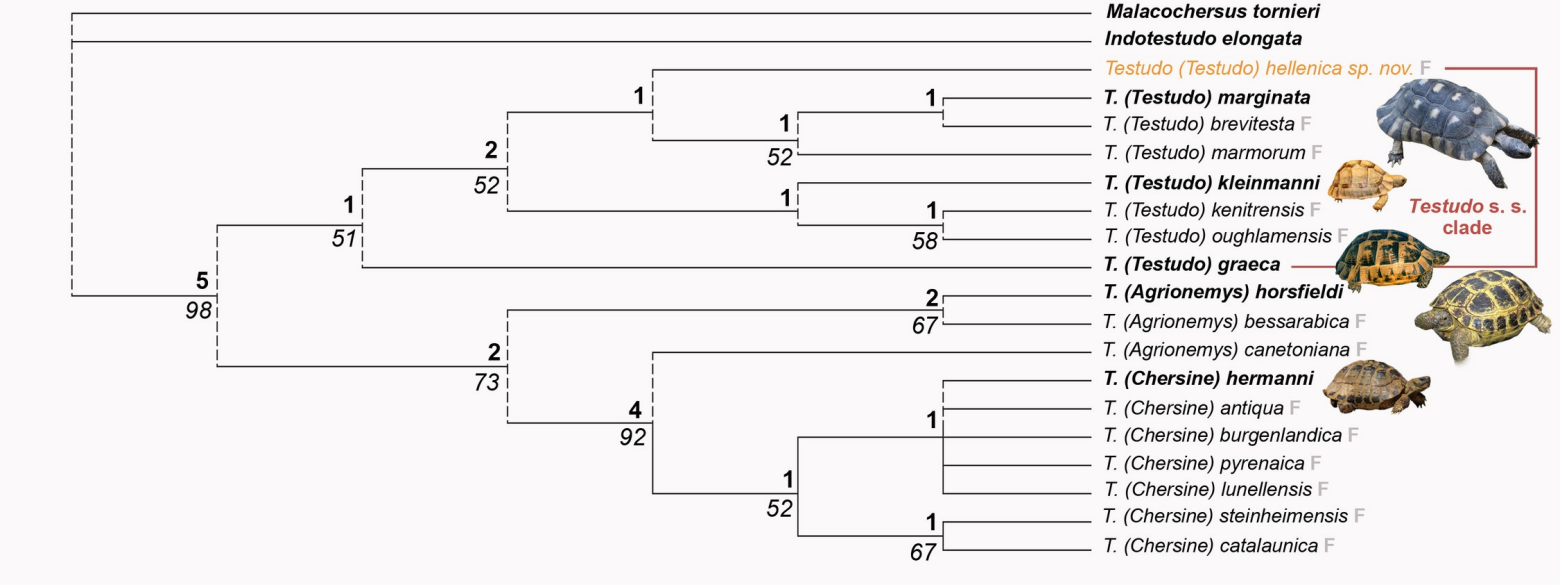
Results of the cladistic analysis corresponding to a strict consensus of 3 equally parsimonious trees of 72 steps (consistency index = 0.61 and retention index = 0.76). The topology of the strict consensus tree obtained is close to the single most parsimonious tree described by Lujan et al. [32] and to the molecular phylogeny tree obtained for the extant taxa [37]. Bremer indices are noted above clades and numbers represents bootstraps values. Extant species are in bold and the fossil species are indicated by an F.

In the cladogram (Fig 7), *T*. *hellenica* is included within the hinged *Testudo* (*T*. s. s.), and this is supported by the angle of the pectoro-abdominal sulcus (11: 1). Moreover, *T*. *hellenica* appears as the sister taxon of the clade formed by the extant *T*. *marginata* and the fossil *T*. *marmorum* and *T*. *brevitesta* (Fig 7). This group is defined by two characters,: a markedly elongated shell contour (15: 2), and a femoro-anal sulcus laterally forming an S-shaped curve, which is very obliquely oriented compared to the axial plane (35: 2). We also note the shared position of the anteriorly reduced part of the femorals with a forwardly protruding V-shape, while the hypo-xiphiplastral hinge slightly juts out beyond the bottom of the inguinal notches. Finally, *T*. *hellenica* displays the transversally widest hinge and abdomino-femoral sulcus, except on the lateral parts, a condition particularly close to that of *T*. *marginata*. The position of *T*. *hellenica* based on this phylogeny analysis is consistent with the late Miocene origin for crown *Testudo*, already suggested by a total evidence analysis of Pan-Testudinidae [11].

## Conclusions

Before this study, the earliest occurrence of a hinged testudinid was *T*. *marmorum*, from the Turolian of Pikermi in Greece (MN 12, around 7.3 Ma [14]). Consequently, *T*. *hellenica* corresponds to the earliest known representative of the *Testudo* s. s. (= hinged *Testudo*) lineage. Its occurrence 9 million years ago in Greece is not unexpected when considering the consistent climatic conditions that prevailed in this region during the late Miocene. The pollen assemblages [43] together with the enamel oxygen stable isotopic analysis [44–48] indicate climatic conditions that were not significantly different from those that are observed today in the southern Balkans, especially in the Axios River Valley. These conditions are characterized by a high seasonal amplitude in terms of temperatures and rainsfalls. Both of these parameters play a primary role in shaping the distribution of *T*. *graeca* [49, 50], particularly at the subspecies level for which some taxa such as *T*. *g*. *marokensis* and *T*. *g*. *cyrenaica* [51] are considered to be dependent on humid environments.

Due to the absence of either well documented or preserved Mediterranean tortoise fossils from the Miocene, the record from RPI in Greece is also significant in providing an additional data for phylogenetic and molecular studies, in particular for understanding the radiation of the evolutionary history of the *Testudo* lineage, a topic which is still debated [40, 52]. Based on the molecular data [7, 39, 40], the estimated divergence of a crown-*Testudo* with a kinetic plastral hinge originated between the late Oligocene and the late Miocene. A more recent total evidence phylogenetic analysis provided an age estimation of around 8 Ma [11] whereas the Vallesian age of *Testudo hellenica* sp. nov. pushes this divergence back to least 9 Ma and provides important evolutionary information of the carapace of *Testudo* s. s. complex. Finally, our phylogenetic analysis suggests that *T*. *marmorum* is closer to *T*. *marginata* (peripheral plates 8–11 posteriorly directed and widened, nuchal plate as long as it is wide) than to *T*. *kleinmanni*, which is more consistent with the fossil record as it notably reduces the ghost lineage for *T*. *marginata*.

## Supporting information

S1 Table Character description of the states employed in the cladistic analysis [30, 32, 38, 53–56]. (DOCX)
