# Supplementary material for: The earliest known crown-Testudo tortoise from the late Miocene (Vallesian, 9 Ma) of Greece
Source: PLoS One. 2020 Apr 8;15(4):e0224783. doi: 10.1371/journal.pone.0224783 (PMC7141670; doi:10.1371/journal.pone.0224783)
Supplement: S1 Table — (DOCX) [file pone.0224783.s001.docx]

**1.** The two suprapygal plates together: 0, do not constitute a trapezoid; 1, constitute a trapezoid. [Modified from [32] and [53]: Ch. 7].

**2.** Contact between marginal scutes and the limits between adjacent pleural scutes: 0, the limits between pleurals only contact odd marginals; 1, the limits between pleurals contact both odd and even marginals. [53].

**3.** Femur (greater and lesser) trochanters: 0, not medially developed, with intertrochanteric fossa visible in ventral view; 1, medially developed, with intertrochanteric fossa invisible in ventral view. [[32], modified from [53]: Ch. 17].

**4.** Hypo-xiphiplastral hinge: 0, absent; 1, present. [[32], modified from [53]: Ch. 3].

**5.** Anterior and posterior borders of marginal scute 5 in lateral view: 0, obliquely directed relative to the anterior and posterior borders of peripheral plates 5–6; 1, parallel to the anterior and posterior borders of peripheral plates 5–6. [32].

**6.** Shell bridge: 0, not elevated (much lower than the marginals and peripherals); 1, elevated (sub-equal or much higher than the marginals and peripherals). [Modified from [32] and [53]: Ch. 1]. The character coding is changed as ? for *T. bessarabica*, as the plastron is lacking in the specimens laterally figured in [54].

**7.** Prootic: 0, well exposed both anteriorly and dorsally; 1, partially concealed anteriorly by the parietal, which only contacts the quadrate anteriorly from the foramen stapedio-temporale; 2, completely concealed dorsally and anteriorly by the parietal, which extensively contacts the quadrate both anteriorly and posteriorly. [[32], modified from [55]].

**8.** Peripheral plates 8–11 in lateral view: 0, more or less ventrally and not posteriorly directed; 1, posteriorly directed. Modified from [32].

**9.** Vertebral scute 3 in dorsal view: 0, at least 1.4 times wider than long; 1, <1.4 wider than long. [32].

**10.** Vertebral scute 5 in dorsal view: 0, not wider than vertebrals 1–4; 1, wider than vertebrals 1–4. [[32], modified from [56]: Ch. 16].

**11.** Pectoro-abdominal sulcus: 0, transversely aligned or only slightly inclined (≤ 18°) relative to horizontal; 1, steeply inclined (> 18°) relative to horizontal. [32].

**12.** Lateral corner of nuchal plate: 0, not covered or only slightly covered by pleural scute 1; 1, markedly covered by pleural scute 1. [[32], modified from [56]: Ch. 4].

**13.** Peripheral plate 1: 0, not markedly protruding anteriorly from the carapace outline; 1, markedly protruding anteriorly from the carapace outline. [32].

**14.** Adult shell shape: 0, low or elevated, but with a flat dorsal surface; 1, elevated, clearly domed and even with a tectiform shape. [Modified from [32] and [53]: Ch. 0].

**15.** Adult shell contour in dorsal view: 0, suboval or quadrangular; 1, slightly elongated (elliptical); 2, markedly elongated; 3, more round with wide rounded borders. [Modified from [32] and [53]: Ch. 0]. The character coding is changed by 2 for *Indotestudo* and 3 for *T. brevitesta*, after the specimens figured in [30].

**16.** Contour of the anterior plastral lobe: 0, rounded (not markedly angulated); 1, trapezoidal (markedly angulated). [32].

**17.** Extensive contact between peripheral plate 6 and hyoplastron: 0, absent (other than a punctual connection with the hyoplastron, it only contacts the hypoplastron); 1, present (contacts both hyo- and hypoplastron). [32].

**18.** Cervical scute: 0, present and well developed dorsally and viscerally; 1, present but dorsally reduced; 2, dorsally absent and viscerally reduced to absent. [[32], modified from [53]: Ch. 13].

**19.** Suprapygal plates: 0, unfused; 1, fused. [[32], modified from [56]: Ch. 12].

**20.** Width of neural plates 2–8: 0, more than twice as wide as long; 1, less than twice as wide as long. [32].

**21.** Supracaudal scute: 0, undivided by a groove; 1, divided by a groove at least dorsally. [[32], modified from [53]: Ch. 16]. *T. pyrenaica* is coded A.

**22.** Posterior widening of the shell behind peripheral plate 7 in dorsal view: 0, absent or only poorly developed; 1, conspicuously present. [32].

**23.** Neural plate series; 0, not reduced (eight neurals); 1, reduced (seven neurals). [[32], modified from [53]: Ch. 5].

**24.** Pygal plate: 0, generally trapezoidal (without anterolateral borders); 1, generally hexagonal (with small anterolateral borders); 2: pentagonal shape. [Modified from [32] and [53]: Ch. 8].

**25.** Dorsal epiplastral lip in medial view: 0, neither posteriorly ascending nor overhanging; 1, posteriorly ascending but not overhanging (without gular pocket); 2, posteriorly ascending but only slightly overhanging (without conspicuous gular pocket); 3, posteriorly ascending and markedly overhanging (with conspicuous gular pocket). [[32], modified from [53]: Ch. 10]

**26.** Ventral relief of the gular scutes: 0, absent (not bulging compared with the humeral scutes); 1, present (at least the anterior portion of the gular scutes). [Modified from [32] and [53]: Ch. 12]. *T. marmorum*: is coded “0” instead of “?” and *T. lunellensis* by a A.

**27.** Vertebral scute 5: 0, heptagonal or hexagonal with sub-equal anterolateral and posterolateral borders; 1, hexagonal with posterolateral borders much shorter than the anterolateral ones, trapezoidal or even flask-shaped (narrow anteriorly). [[32], modified from [38]: Ch. 18].

**28.** Pygal plate in lateral view: 0, posteroventrally directed; 1, posteriorly directed. [32].

**29.** Outline of the nuchal plate: 0, hexagonal; 1, octagonal. [[32], taken from [55]: Ch. 1; [30]: Ch. 1].

**30.** Protrusions on the peripherals (at the limit with the sulci between marginals): 0, absent or poorly developed; 1, well developed. [[32], taken from [30]: Ch. 20].

**31.** Angle between the gulo-humeral sulcus and the sagittal plane: 0, ≥ 45; 1, < 45°. [[32], modified from [30]: Ch. 32].

**32.** Medial length of humeral scute relative to the medial length of gular scute: 0, humeral not longer than gular; 1, humeral longer than gular. [[32], taken from [30]: Ch. 33].

**33.** Position of the humeropectoral sulcus: 0, posterior to the entoplastron; 1, medially coinciding with the posterior suture of the entoplastron; 2, crossing the entoplastron. [[32], taken from [30]: Ch. 34].

**34.** Shape of the humeropectoral sulcus: 0, curved and obliquely orientated relative to the sagittal plane; 1, rather straight and approximately perpendicular to the sagittal plane. [[32], modified from [30]: Ch. 35].

**35.** Shape of the femoro-anal sulcus: 0, straight or only slightly curved, and obliquely orientated (forming an angle < 45°) relative to the axial plane; 1, slight or only slightly curved, and roughly parallel to the axial plane (i.e. perpendicular to the sagittal plane); 2, laterally forming an S-shaped curve, and very obliquely orientated (forming an angle ≥ 45°) relative to the axial plane. [[32], modified from [30]: Ch. 38].

**36.** Medial length of the anal scute relative to the medial length of the femoral scute: 0, anal shorter than femoral; 1, anal longer than femoral. [[32], taken from [54]: Ch. 44; [30]: Ch. 39].

**37.** Shape of the nuchal plate: 0, markedly wider than long; 1, as long as it is wide or only slightly wider than long. [[32], taken from [30]: Ch. 2].
